# Perianal Basal Cell Carcinoma Successfully Managed with Excisional Biopsy

**DOI:** 10.1155/2019/6268354

**Published:** 2019-06-12

**Authors:** Paola C. Aldana, Harris G. Yfantis, Preeti R. John

**Affiliations:** ^1^University of Maryland School of Medicine, Baltimore, MD, USA; ^2^Baltimore Veterans Affairs Medical Center, Baltimore, MD, USA

## Abstract

Basal cell carcinoma (BCC) is the most common cutaneous malignancy in the United States and is often nonaggressive. Its location in the perianal region is very rare and it is estimated that only 0.08% of all BCCs occur in this region. Herein, we present a case of perianal basal cell carcinoma, nodular type. The diagnosis was made using excisional biopsy of a skin lesion. Immunohistochemical staining confirmed the diagnosis: it showed diffuse and strong positivity for smooth muscle actin (SMA) and monoclonal antibody BER-Ep4 and was negative for carcinoembryonic antigen (CEA), pancytokeratin (AE1/AE3), and epithelial membrane antigen (EMA). The treatment of choice has traditionally been local excision to clear margins but the newest guidelines recommend Mohs Micrographic surgery (MMS) or standard 4mm surgical margins for this high-risk BCC. Our patient was successfully treated using excisional biopsy without recurrence. In select patients with lesions smaller than 1cm, excisional biopsy may be sufficient to treat the disease and may be better tolerated than MMS and wider surgical margins. Literature review suggests a predisposition for perianal BCC in individuals susceptible to cutaneous malignancies. Therefore, any history of cutaneous malignancy should further prompt clinicians to examine nonsun exposed areas on full body skin exams.

## 1. Introduction

Basal cell carcinoma (BCC) is the most common cutaneous malignancy in the United States and mostly frequently occurs on sun exposed areas such as the head and neck [1]. Although it can occur on other areas, involvement of the perianal skin and genitals is extremely rare [2]. It is estimated that perianal BCC accounts for only 0.08% of all BCC and 0.2% of all anorectal neoplasms [1, 3]. In fact, there are less than 200 cases of BCC on the perianal skin and genitals reported in the literature [1]. We present a case of nodular perianal BCC that was diagnosed using immunohistochemical staining, for which excisional biopsy was performed with no subsequent recurrence.

## 2. Case Presentation

An 89-year-old male with no history of cutaneous malignancies was referred to General Surgery for evaluation of a persistent nodule on the skin of the right perianal region that he had noticed for more than a year. Past medical history was significant for gastric lymphoma (non-Hodgkins follicular type, with spontaneous remission).

He palpated the perianal lesion while cleaning the skin in this region. The lesion was not associated with bleeding, oozing, pain, or itching. He denied any concomitant skin lesion or rashes. He also denied fever, chills, or abdominal symptoms. On physical exam, a single small 0.5 cm cutaneous nodule with central dimpling and no discoloration was visualized in the perianal region. No concerning lesions were palpated on digital rectal exam. An excisional biopsy of the nodule was performed for histopathological examination.

**Figure 1:**
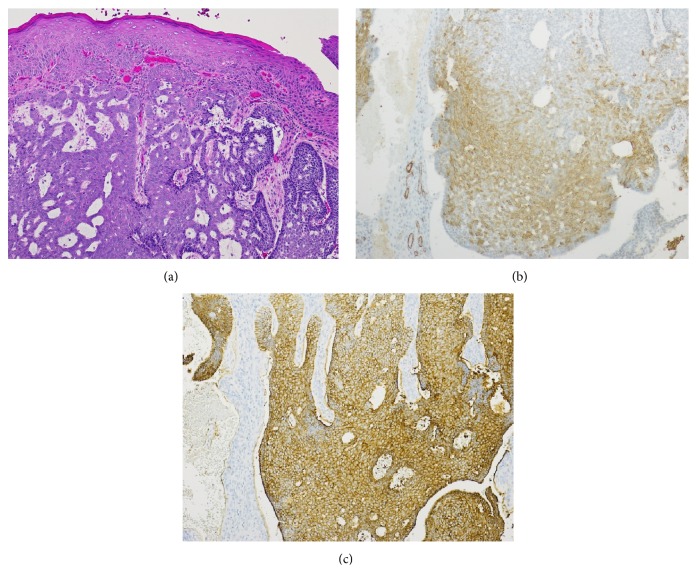
*Histopathological analysis of perianal nodule biopsy. *(a) Hematoxylin and Eosin (H&E) stain (original magnification 100x) showing anastomosing cords of basaloid cells with overlying connection to epidermis and stromal retraction. (b) Immunohistochemical staining for smooth muscle actin (original magnification 100x) showing diffuse, strong positivity. (c) Immunohistochemical staining for Ber-EP4 (original magnification 100x) showing diffuse, strong, membranous positivity.

Microscopic exam confirmed the diagnosis of perianal BCC, nodular type (Figure 1(a)). Histopathology revealed a lesion strongly and diffusely positive for immunohistochemical staining of smooth muscle actin (SMA) (Figure 1(b)) and negative for carcinoembryonic antigen (CEA) and pancytokeratin (AE1/AE3), supporting the diagnosis. Staining for epithelial membrane antigen (EMA) was negative in the tumor cells while monoclonal antibody BER-Ep4 displayed strong and diffuse positivity, further supporting the diagnosis (Figure 1(c)). The patient responded well to excision of the lesion and no further treatment was required.

## 3. Discussion

Basal cell carcinoma of the perianal skin is a rare entity that is most frequently reported by individual case reports or case series. There is a male predominance and the mean age of onset is 67 years [4]. It may vary in its clinical presentation from erythematous papules and patches to nodules, plaques, and ulcers that may present with bleeding, pain, itching, and mucoid discharge [1, 5]. The majority of these lesions are smaller than 2cm and are located* outside* the anal verge [4]. This location may be useful in distinguishing perianal BCC from basaloid squamous cell carcinoma (BSCL). BSCL is a common anal tumor and most commonly occurs in the anal canal or anorectum (*inside* the anal verge) [6]. Although several subtypes of BCC exist, the nodular type is the most common in the perianal region [6].

The etiology of perianal BCC remains unknown; however chronic irritation, trauma, immunosuppressive medications, and radiation have been suggested as causative factors [1]. Although most patients with perianal BCC do not have a personal history of tanning bed use or nude sunbathing, approximately 1/3 have a history of skin cancer in sun exposed areas [1, 3]. This association suggests a predisposition in individuals susceptible to cutaneous malignancies. Therefore, any history of cutaneous malignancy should further prompt clinicians to examine nonsun exposed areas on full body skin exams. Furthermore, frequent BCC development has been associated with an increased overall cancer risk; therefore occurrence of BCC more than once in the same patient should prompt close surveillance for other malignancies (our patient had a history of gastric lymphoma) [7].

It is important to obtain histologic verification of the disease in order to distinguish it from more aggressive malignancies with higher capacity for metastasis such as BSCL. The classic histology of perianal BCC reveals peripheral palisading and peritumoral slits [4]. Immunohistochemical staining is also useful as perianal BCC usually displays diffuse and strong staining for SMA and Ber-EP4 [6, 8, 9], while BSCL does not [9]. Positive immunohistochemical reactivity with pancytokeratin, EMA, and CEA is not consistent with perianal BCC and may suggest a diagnosis of BSCL [9].

According to The National Comprehensive Cancer Network, a BCC in the perianal region is considered ‘high-risk' and Mohs Micrographic surgery (MMS) is recommended, although standard 4mm margin excision can be considered for select tumors [10]. The treatment of choice for perianal BCC has traditionally been local excision to clear margins, especially for lesions that are less than 2cm [4]. Local excision has proven to be the extremely successful since there is usually no recurrence of disease [1, 4]. Larger lesions may require skin grafting for closure and only those involving the anal canal or extending into the surrounding tissues require abdominoperineal resection of the rectum [4, 5]. Our patient exhibited a lesion that was 0.5cm in size and excisional biopsy was sufficient treatment for his disease.

Since perianal BCC is so uncommon and is located in an inconspicuous skin area, it may be easily overlooked by clinicians. Therefore, we recommend that gluteal and genital areas be included in full body skin exams, particularly in higher risk individuals. Suspicious lesions should be biopsied and immunohistochemical staining performed to establish the diagnosis.

In summary, perianal BCC is a nonaggressive malignancy with excellent prognosis following local excision to clear margins. Specifically, for perianal lesions smaller than 1cm, excisional biopsy may be sufficient and better tolerated than MMS or standard local excision with 4mm margins. This case report supports the notion that proper clinical vigilance and early detection of perianal BCC can result in excellent prognosis.
